# Supplementary material for: Policies to Create Healthier Food Environments in Canada: Experts’ Evaluation and Prioritized Actions Using the Healthy Food Environment Policy Index (Food-EPI)
Source: Int J Environ Res Public Health. 2019 Nov 14;16(22):4473. doi: 10.3390/ijerph16224473 (PMC6888279; doi:10.3390/ijerph16224473)
Supplement: Supplementary file 1 [file ijerph-16-04473-s001.pdf]

International Journal of *Environmental Research and Public Health*

MDPI

Supplemental Material

Policies to create healthier food environments in Canada: Experts' evaluation and prioritized actions using the Healthy Food Environment Policy Index

Table S1. Indicators and good practice statements used in the Canadian Food-EPI per governmental jurisdiction, Food-EPI Canada, 2017.

Food-EPI Indicator	Abbreviated Indicator name	Abbreviated Good Practice Statement	Regulatory Jurisdiction in Canada	
			Federal	Provincial/ Territorial
		**Policy Component**		
COMP1	Composition targets for packaged foods	Food composition targets for processed foods for nutrients of concern in certain foods or food groups are in place	X	
COMP2	Composition targets for out-of-home foods	Food composition targets for out-of-home meals in food service outlets for nutrients of concern in certain foods or food groups are in place	X	X
LABEL1	Nutrition information on labels	Ingredient lists and nutrient declarations (including warning labels) in line with Codex recommendations are present on the labels of all packaged foods	X	
LABEL2	Health claim regulations	Regulatory systems are in place for approving/reviewing claims on foods to protect consumers against unsubstantiated and misleading nutrition and health claims	X	
LABEL3	Front-of-package food labelling	A single, consistent, interpretive, evidence-informed front-of-pack (FOP) supplementary nutrition information system is applied to all packaged foods	X	
LABEL4	Menu labelling	A consistent, single, simple, clearly-visible system of labelling the menu boards of all quick service restaurants (e.g., fast food chains) is applied by the government, which allows consumers to interpret the nutrient quality and/or energy content of foods and meals	X	X
PROMO1	Promotion to children via broadcast media	Policies restrict exposure and power of promotion of unhealthy foods to children through broadcast media (TV, radio)	X	X
PROMO2	Promotion to children via non-broadcast media	Policies restrict exposure and power of promotion of unhealthy foods to children through non-broadcast media (e.g. Internet, social media, food packaging, sponsorship, outdoor and public transport advertising)	X	X
PROMO3	Promotion to children in children's settings	Policies restrict promotion of unhealthy foods to children in settings where children gather (e.g. preschools, schools, sport and cultural events)	X	X
PRICE1	Minimize taxes on healthy foods	Taxes on healthy foods are minimised to encourage healthy food choices where possible	X	X

PRICE2	Increase taxes on unhealthy foods	Taxes on unhealthy foods are in place and increase the retail prices of these foods by at least 10% to discourage unhealthy food choices where possible, and these taxes are reinvested to improve population health	X	X
PRICE3	Subsidies on foods	Existing subsidies on foods favour healthy rather than unhealthy foods	X	X
PRICE4	Food-related income support	The government ensures that food-related income support programs are for healthy foods	X	X
PROV1	School nutrition policies	Clear, consistent policies in schools and early childhood education and care services for food service activities provide and promote healthy food choices	X	X
PROV2	Public sector nutrition policies	Clear, consistent policies in public sector settings for food service activities provide and promote healthy food choices	X	X
PROV3	Support for nutrition policies	Good support and training systems to help schools and other public sector organisations and their caterers meet the healthy food service policies and guidelines		X
PROV4	Private company nutrition policies	Government actively encourages and supports private companies to provide and promote healthy foods and meals in their workplaces	X	X
RETAIL1	Planning policies for unhealthy food outlets	Zoning laws provide robust mechanisms to place limits on the density or placement of outlets selling mainly unhealthy foods in communities	X	X
RETAIL2	Planning policies for healthy food outlets	Zoning laws provide robust mechanisms to encourage the availability of outlets selling fresh fruit and vegetables	X	X
RETAIL3	Food availability in food stores	Support systems encourage food stores to promote the in-store availability of healthy foods and to limit the in-store availability of unhealthy foods	X	X
RETAIL4	Food availability and promotion in restaurants	Support systems encourage food service outlets to increase the promotion and availability of healthy foods and to decrease the promotion and availability of unhealthy foods	X	X
TRADE1	Risk impact assessments	Risk impact assessments before and during the negotiation of trade and investment agreements identify and evaluate the impacts of agreements on population nutrition and health	X	
TRADE2	Manage and protect regulatory capacity	The government adopts measures to manage investment and protect their regulatory capacity with respect to public health nutrition	X	
		**Infrastructure Support Component**		
LEADERSHIP1	Political support	There is strong, visible, political support for improving food environments, population nutrition, diet-related NCDs and related inequalities	X	X
LEADERSHIP2	Population intake targets	Clear population intake targets for the nutrients of concern have been established	X	X
LEADERSHIP3	Dietary guidelines	Clear, interpretive, evidence-informed food-based dietary guidelines have been established and implemented	X	
LEADERSHIP4	Implementation plan to improve food environments	There is a comprehensive, transparent, up-to-date implementation plan to improve food environments, reduce the intake of the nutrients of concern, and reduce diet-related NCDs	X	X
LEADERSHIP5	Priorities for inequalities	Government priorities to reduce inequalities or protect vulnerable populations in relation to diet, nutrition, obesity and NCDs have been established	X	X

GOVERNANCE1	Restrict commercial influence	Procedures to restrict commercial influences on the development of policies related to food environments where they have conflicts of interest are in place	X	X
GOVERNANCE2	Evidence in policymaking	Policies and procedures for using evidence in the development of food policies are in place	X	X
GOVERNANCE3	Transparency in policy development	Policies and procedures are implemented for ensuring transparency in the development of food policies	X	X
GOVERNANCE4	Public access to information	The government ensures public access to comprehensive information and key documents (e.g. budget documents, annual performance reviews and health indicators) related to public health nutrition and food environments	X	X
MONIT1	Monitoring food environments	Monitoring systems regularly monitor food environments	X	X
MONIT2	Monitoring population intakes	There is regular monitoring of adult and childhood nutrition status and population intakes against specified intake targets or recommended daily intake levels	X	X
MONIT3	Monitoring overweight and obesity	There is regular monitoring of adult and childhood overweight and obesity prevalence using anthropometric measurements	X	X
MONIT4	Monitoring NCD prevalence and risk factors	There is regular monitoring of the prevalence of NCD risk factors and occurrence rates (e.g. prevalence, incidence, mortality) for the main diet-related NCDs	X	X
MONIT5	Evaluation of programs and policies	Evaluation of major programs and policies to assess effectiveness and contribution to achieving the nutrition and health goals	X	X
MONIT6	Monitoring health inequalities	Regular monitoring of progress towards reducing health inequalities or health impacts in vulnerable populations and social determinants of health	X	X
FUNDING1	Sufficient population nutrition budget	The ‘population nutrition’ budget, as a proportion of total health spending and/or in relation to the diet-related NCD burden is sufficient to reduce diet-related NCDs	X	X
FUNDING2	Government-funded research	Government funded research is targeted for improving food environments, reducing obesity, NCDs and their related inequalities	X	X
FUNDING3	Health promotion agency	There is a statutory health promotion agency in place that includes an objective to improve population nutrition	X	X
PLATFORMS1	Coordination mechanism across government	Coordination mechanisms across departments and levels of government (national and state) to ensure policy coherence, alignment, and integration of food-related policies across governments	X	X
PLATFORMS2	Coordination mechanism with commercial food sector	There are formal platforms between government and the commercial food sector to implement healthy food policies	X	X
PLATFORMS3	Coordination mechanism with civil society	There are formal platforms for regular interactions between government and civil society on food policies and other strategies to improve population nutrition	X	X
PLATFORMS4	Systems-based approach with local organizations	Broad, coherent, effective, integrated and sustainable systems-based approach with local organisations to improve food environments at a national level	X	

HIAP1	Health considerations in all food policies	Population nutrition, health outcomes and reducing health inequalities or health impacts in vulnerable populations are considered and prioritised in the development of all government policies relating to food	X	X
HIAP2	Health impact assessments in non-food policies	There are processes (e.g. HIAs) to assess and consider health impacts during the development of other non-food policies	X	X

For each indicator, you will see a screen like this:

POLICY AREA: FOOD COMPOSITION

Food-EPI vision statement: There are government systems implemented to ensure that, where practicable, processed foods and out-of-home meals minimize the energy density and the nutrients of concern (salt, saturated fat, trans fat, added sugar)

The **Food-EPI vision statement** is a theoretical vision for what we might hope policy can achieve in each policy domain.

COMP2. Food composition targets/standards/restrictions for out-of-home meals

Food-EPI good practice statement: *The government has established food composition targets/standards for out-of-home meals in food service outlets for the content of the nutrients of concern in certain foods or food groups if they are major contributors to population intakes of these nutrients of concern (trans fats, added sugars, salt, saturated fat)*

This is the definition of the good practice indicator you will be rating

INTERNATIONAL EXAMPLES:

- **New York City, USA**: In 2006, New York City's Health Code was amended to restrict the amount of *trans*-fats to a maximum of 0.5g in food served by all food service establishments. Violators are subject to fines up to $2,000.00. Other US cities have since banned restaurants from serving *trans*-fats.
- **New York City, USA**: In 2009, New York City established voluntary salt guidelines for various restaurant and store-bought foods, which evolved into the National Salt Reduction Initiative to reduce excess sodium by 25% in packaged and restaurant foods.
- **New Zealand**: In New Zealand, The Chip group, funded 50% by the Ministry of Health and 50% by industry, set an industry standard for deep frying oils to maximum 28% saturated fat, 3% linoleic acid and 1% of *trans*-fat for deep-fried chips.
- **The Netherlands**: In 2014, the Dutch Ministry of Health, Welfare and Sport signed an agreement with trade organizations representing food manufacturers, supermarkets, hotels, restaurants, caterers and the hospitality industry to lower the levels of salt, saturated fat, calories in food products and increase the healthiness of the food supply by 2020.

These are the international best practice examples or benchmarks where countries are doing particularly well in policy areas. This is what you will rate the provincial policy against.

CONTEXT:
While regulations for packaged food are primarily based at the federal level, composition targets or standards for restaurant foods can potentially fit within the mandate of provincial or territorial governments.

POLICY DETAILS:
There are no food standards for out-of-home meals at the provincial level in Ontario.

This is a summary of any relevant context and the current evidence of implementation in the province.

Figure S1. Image of the introductory screen provided for online ratings, Food-EPI Canada, 2017.

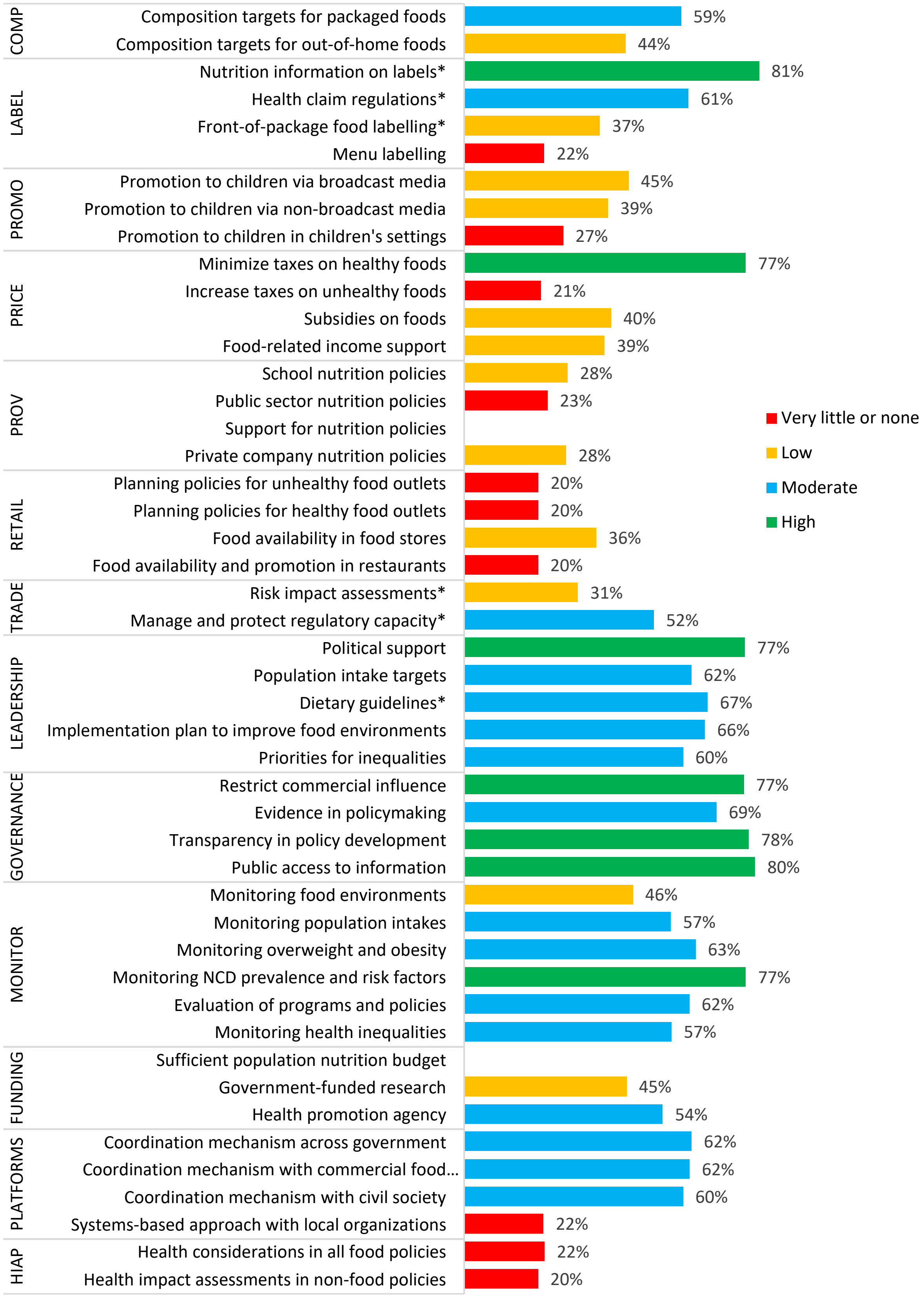

* indicates indicators that were only rated at the federal level

Figure S2. Ratings for federal government only for 45 indicators, Food-EPI Canada, 2017.

Indicator	AB	BC	MB	NB	NL	NS	NWT	ON	PEI	QC	SK	YK
Composition targets for out-of-home foods	33%	64%	20%	20%	20%	20%	20%	20%	20%	54%	20%	20%
Menu labelling	20%	44%	24%	20%	20%	20%	20%	83%	20%	20%	20%	20%
Promotion to children via broadcast media	20%	23%	20%	32%	20%	20%	20%	23%	20%	97%	20%	20%
Promotion to children via non-broadcast media	26%	20%	20%	20%	20%	20%	20%	23%	20%	87%	20%	20%
Promotion to children in children's settings	20%	27%	28%	56%	27%	57%	20%	20%	50%	80%	25%	20%
Minimize taxes on healthy foods	83%	83%	83%	83%	83%	83%	83%	83%	83%	83%	83%	83%
Increase taxes on unhealthy foods	20%	20%	20%	20%	20%	20%	33%	20%	20%	49%	20%	33%
Subsidies on foods	49%	40%	55%	20%	27%	47%	80%	54%	20%	73%	20%	30%
Food-related income support	40%	40%	40%	44%	57%	30%	26%	43%	27%	57%	28%	20%
School nutrition policies	74%	77%	80%	80%	57%	87%	80%	77%	47%	63%	52%	40%
Public sector nutrition policies	69%	73%	32%	52%	50%	70%	47%	46%	24%	74%	24%	37%
Support for nutrition policies	86%	73%	76%	76%	73%	83%	80%	83%	57%	54%	36%	43%
Private company nutrition policies	73%	63%	32%	36%	47%	20%	20%	33%	37%	43%	20%	44%
Planning policies for unhealthy food outlets	40%	28%	50%	28%	23%	27%	20%	23%	23%	23%	20%	27%
Planning policies for healthy food outlets	23%	23%	20%	44%	50%	20%	23%	20%	20%	20%	20%	20%
Food availability in food stores	20%	20%	20%	28%	43%	20%	20%	20%	20%	57%	20%	20%
Food availability and promotion in restaurants	20%	20%	20%	20%	20%	23%	20%	20%	20%	46%	20%	20%
Political support	31%	37%	20%	45%	57%	30%	51%	51%	30%	43%	20%	20%
Population intake targets	43%	56%	44%	44%	53%	30%	34%	37%	30%	31%	20%	40%
Strategy/plan to improve food environments	34%	73%	52%	52%	70%	67%	31%	83%	56%	49%	32%	63%
Priorities for inequalities	31%	27%	65%	68%	80%	37%	54%	46%	40%	57%	20%	57%
Restrict commercial influence	71%	63%	40%	48%	63%	87%	54%	60%	43%	53%	24%	60%
Evidence in policymaking	51%	20%	56%	36%	67%	23%	20%	60%	23%	29%	20%	33%
Transparency in policy development	43%	57%	40%	70%	73%	43%	60%	80%	40%	47%	20%	20%
Public access to information	91%	91%	91%	91%	91%	91%	91%	91%	91%	91%	91%	91%
Monitoring food environments	51%	20%	20%	48%	37%	20%	20%	20%	23%	63%	40%	27%
Monitoring population intakes	70%	67%	60%	80%	50%	43%	20%	73%	53%	37%	52%	47%
Monitoring overweight and obesity	63%	43%	48%	68%	50%	43%	51%	54%	30%	63%	68%	47%
Monitoring NCD prevalence and risk factors	83%	70%	56%	84%	67%	60%	43%	77%	67%	71%	68%	63%
Evaluation of programs and policies	34%	37%	32%	60%	57%	40%	20%	33%	33%	63%	20%	20%
Monitoring health inequalities	63%	73%	76%	88%	60%	20%	26%	60%	63%	57%	28%	20%
Sufficient population nutrition budget												
Government-funded research	87%	27%	28%	65%	56%	50%	20%	49%	40%	63%	20%	20%
Health promotion agency	31%	27%	20%	76%	35%	36%	20%	86%	35%	80%	33%	23%
Coordination mechanism across government	29%	47%	50%	96%	88%	50%	63%	60%	24%	74%	44%	27%
Coordination mechanism w/ food sector	30%	20%	52%	25%	47%	20%	20%	20%	20%	69%	20%	37%

Coordination mechanism with civil society	20%	20%	72%	84%	77%	20%	46%	68%	30%	77%	20%	37%
Health considerations in all food policies	51%	20%	30%	25%	27%	33%	20%	57%	33%	86%	20%	24%
Health impact assessments in non-food policies	49%	50%	20%	28%	43%	36%	20%	51%	40%	91%	20%	20%

Table S2. Ratings for provincial and territorial governments for 38 indicators, Food-EPI Canada, 2017.
